# Lubiprostone Improves Distal Segment-Specific Colonic Contractions through TRPC4 Activation Stimulated by EP3 Prostanoid Receptor

**DOI:** 10.3390/ph17101327

**Published:** 2024-10-04

**Authors:** Byeongseok Jeong, Jun Hyung Lee, Jin-A Lee, Seong Jung Kim, Junhyung Lee, Insuk So, Jae Yeoul Jun, Chansik Hong

**Affiliations:** 1 Department of Physiology, Chosun University College of Medicine, Gwangju 61452, Republic of Korea; ggb01099@snu.ac.kr (B.J.); klio0427@chosun.ac.kr (J.-A.L.); jun919792@gmail.com (J.L.); jyjun@chosun.ac.kr (J.Y.J.); 2 Department of Physiology, Seoul National University College of Medicine, Seoul 03080, Republic of Korea; insuk@snu.ac.kr; 3 Department of Internal Medicine, Chosun University College of Medicine, Gwangju 61452, Republic of Korea; pp3614@chosun.ac.kr (J.H.L.); ygegh@chosun.ac.kr (S.J.K.)

**Keywords:** lubiprostone, TRPC4 channel, EP receptors, smooth muscle contraction, colonic motility

## Abstract

Background: Prokinetic agents are effective in increasing gastrointestinal (GI) contractility and alleviating constipation, often caused by slow intestinal motility. Lubiprostone (LUB), known for activating CLC-2 chloride channels, increases the chloride ion concentration in the GI tract, supporting water retention and stool movement. Despite its therapeutic efficacy, the exact mechanisms underlying its pharmacological action are poorly understood. Here, we investigated whether LUB activates the canonical transient receptor potential cation channel type 4 (TRPC4) through stimulation with E-type prostaglandin receptor (EP) type 3. Methods: Using isotonic tension recordings on mouse colon strips, we examined LUB-induced contractility in both proximal and distal colon segments. Quantitative real-time polymerase chain reaction (qRT-PCR) was conducted to determine mRNA levels of EP1-4 receptor subtypes in distal colonic muscular strips and isolated myocytes. The effects of a TRPC4 blocker and EP3 antagonist on LUB-stimulated contractions were also evaluated. Results: LUB showed significant contraction in the distal segment compared to the proximal segment. EP3 receptor mRNA levels were highly expressed in the distal colon tissue, which correlated with the observed enhanced contraction. Furthermore, LUB-induced spontaneous contractions in distal colon muscles were reduced by a TRPC4 blocker or EP3 antagonist, indicating that LUB-stimulated EP3 receptor activation may lead to TRPC4 activation and increased intracellular calcium in colonic smooth muscle. Conclusions: These findings suggest that LUB improves mass movement through indirect activation of the TRPC4 channel in the distal colon. The segment-specific action of prokinetic agents like LUB provides compelling evidence for a personalized approach to symptom management, supporting the defecation reflex.

## 1. Introduction

Recent epidemiological studies have revealed that functional gastrointestinal disorders (FGIDs) affect approximately 15.3% of the adult population [1,2]. The exact etiology of constipation, which is multifactorial and elusive, requires classification based on discernible symptoms and causes [3,4]. Slow transit constipation (STC), which accounts for approximately 55% of constipation cases, is characterized primarily by reduced intestinal motility and delayed transit of intestinal contents. Therefore, STC prescription generally increases intestinal motility with over-the-counter laxatives, such as bulk-forming agents, stimulants, osmotic laxatives, and stool softeners [4,5]. For patients whose symptoms do not improve despite these interventions, prescription drugs are considered, ranging from linaclotide (Linzess^®^), an activator of the guanylate cyclase C receptor in intestinal epithelial cells, to prucalopride (Resolor^®^), a 5-HT4 receptor agonist, and including lubiprostone (LUB; Amitiza^®^), a selective chloride channel activator. Additionally, while the pharmacological agents used in the treatment of constipation are effective in managing symptoms, they are not devoid of side effects. Over-the-counter laxatives, including bulk-forming agents, can cause bloating and abdominal discomfort, while stimulant laxatives may lead to decreased bowel sensitivity and potential dependency when used long-term [5,6]. Osmotic laxatives, although generally safe, can occasionally result in dehydration and electrolyte imbalances. Moreover, prescription drugs such as linaclotide and LUB are associated with risks of severe diarrhea and nausea, emphasizing the need for careful patient monitoring and dosage adjustments. Understanding these adverse effects is crucial for optimizing treatment outcomes and ensuring patient safety.

Consideration of first-line drugs for STC focuses on understanding the complex etiology of these conditions and developing symptomatic prescriptions for patients. Among the first-line drugs, LUB is an osmotic laxative extensively utilized in the management of chronic idiopathic constipation, opioid-induced constipation, and irritable bowel syndrome with constipation [7,8]. The primary therapeutic effect of LUB is attributed to the activation of CLC-2 chloride channels, which are strategically localized in the apical membrane of gastrointestinal (GI) epithelial cells [9]. CLC-2 activation catalyzes a significant increase in the transmembrane secretion of chloride ions into the intestinal lumen [10,11]. Accordingly, water secretion to maintain osmotic balance increases the fluidity of the luminal contents, thereby facilitating bowel motility and easing stool passage.

Additionally, LUB, a prostaglandin derivative, enhances smooth muscle contraction in the GI tract by directly exposing and interacting with the interstitial cells of Cajal (ICCs) [12] and colonic muscular layers [13,14]. Regardless of the previously known effects of CLC-2 activation, recent studies have revealed the role of E-series prostanoid (EP) receptors in the intestinal muscles and enteric plexus, highlighting the efficacy of LUB in treating irritable bowel syndrome and STC [15]. PGE2 functions across inflammation, pain modulation, and regulation of fluid secretion and motility through the activation of EP receptors in the GI tract [15,16,17,18]. EP receptors, categorized into EP1, EP2, EP3, and EP4 subtypes, induce protection against gastric and duodenal damage via EP1 and EP3/EP4 activation, respectively, and promote mucosal healing and angiogenesis through EP4 receptor stimulation. The intracellular sub-signaling pathways of these EP receptors are as follows: EP1 with Gq, EP3 with Gi, and EP2 and EP4 with the Gs pathway. In particular, the Gi and Gq pathways are associated with elevated intracellular Ca^2+^ and reduced cAMP levels, both of which are known to induce smooth muscle contraction. Moreover, Gi- and Gq-coupled receptors increase membrane potential and promote Ca^2+^ influx through the canonical transient receptor potential cation channel type 4 (TRPC4) in GI smooth muscle cells [19,20]. The TRPC4 channel is prominently expressed in the smooth muscles of the GI tract and evokes a muscarinic cation current (m*I*_cat_), which is crucial for myocyte contraction [21,22]. TRPC4 activation, which increases the membrane potential and calcium influx, is critical for muscular strip contractions and GI motility. Notably, our group reported that TRPC4 is activated by Gi-coupled receptors, including muscarinic receptor type 2 and opioid receptors, which are modulated in the GI tract [23,24].

This research focused on elucidating the mechanisms by which LUB enhances colonic motility. We investigated the possibility that the therapeutic actions of LUB extend beyond its known effect of activating CLC-2 chloride channels, suggesting that it also facilitates smooth muscle contraction by activating TRPC4 channels through interactions with EP receptors in myocytes. Additionally, our study examined the expression of EP receptors across various colonic segments to assess the specific responses induced by LUB, providing deeper insights into its therapeutic potential for managing FGIDs.

## 2. Result

### 2.1. Lubiprostone Increases Tonic Contraction of Distal Colonic Muscle Strips in Mice

**Figure 1:**
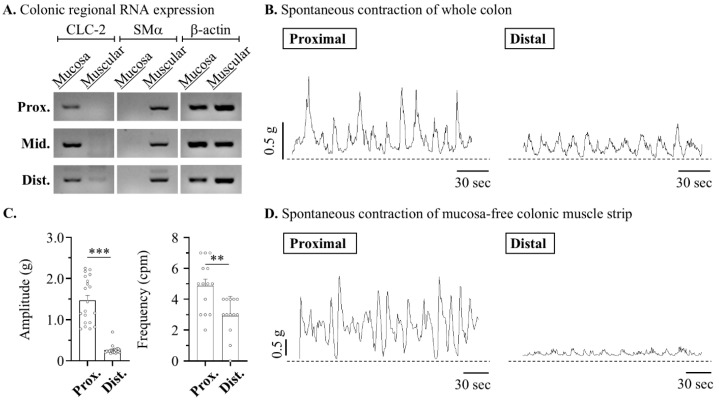
Lubiprostone enhances the amplitude of the contractile wave in the muscle from murine distal colon. The contractile dynamics in proximal and distal segments of mouse colonic strip. (**A**) Representative blots of reverse transcription PCR in mucosa and muscle layer of mouse colon. (**B**,**D**) Representative traces of the circular muscle strips in mouse colon. (**C**) Summarized bar graph of amplitude (left) and frequency (right) of peristaltic waves in mucosa-free colonic muscle strip: proximal (*n* = 14) and distal (*n* = 12). ** *p* < 0.05 and *** *p* < 0.001.

The primary site of the LUB mechanism is the mucosa, which does not necessarily function directly in the smooth muscles of the GI layer. LUB induces contractions by activating CLC-2, a key target protein that increases moisture in the lumen, thereby enhancing muscle motility First, to clarify the target layer of the motility mechanism of the LUB, the expression of CLC-2 in the isolated mucosa and muscle layers was observed. As expected, the RNA expression of CLC-2 was not observed throughout the colonic layer in the isolated muscular strips (Figure 1A). Therefore, we propose that CLC-2 activation is independent of the physiological mechanisms of the muscle layer, and we confirmed the enhancement of colonic motility by LUB treatment. Before this, we had to distinguish between the basal contractions of the proximal and distal colonic segments. The contractile wave is anatomically and functionally distinguished in each colonic segment [25,26]. The proximal colon, contiguous with the small intestine, is adapted for water and electrolyte absorption and is characterized by segmental contractions that facilitate luminal mixing. Conversely, the distal colon, which is proximal to the rectum, specializes in fecal storage and demonstrates an enhanced capability for mass movements that propel stool towards the rectum for evacuation. Therefore, Therefore, the fundamental differences in contractions between the proximal and distal colons were compared, with the colons mounted vertically in an organ bath to measure the tension within the circular muscle layers. As shown in Figure 1B,D, both layers exhibited more elevation in amplitude in the proximal segment; specifically, the amplitude in the proximal segment (0.76 ± 0.09 g) was more elevated than the distal segment (0.10 ± 0.09 g) in the mucosa-free muscular layer (Figure 1C). Additionally, the frequencies of basal spontaneous contractions were observed to show a clear difference, with the distal segment exhibiting 4.86 cycle /min (cpm) compared to 2.92 cpm in the proximal segment.

**Figure 2:**
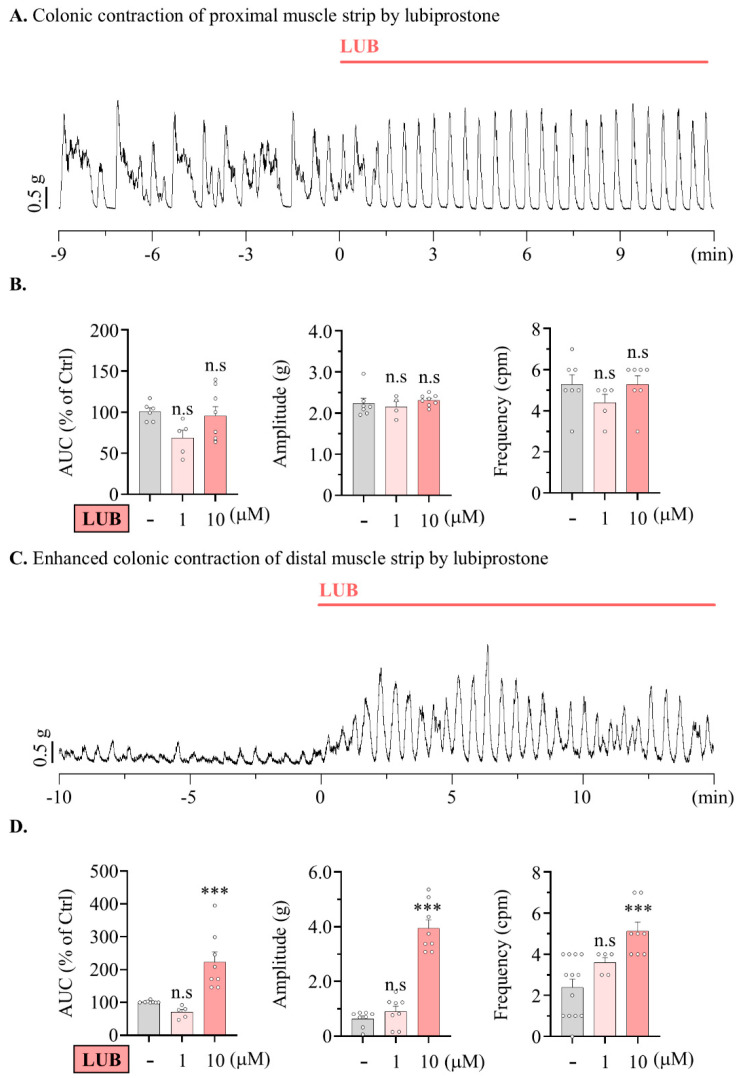
Lubiprostone enhances the amplitude of the contractile wave in mouse distal colonic muscle strip. Lubiprostone enhances the amplitude of the contractile wave in the muscle from nurine distal colon. (**A**,**C**) Representative traces of contractile wave by 10 μM LUB. (**A**) Proximal colonic muscle strip. (**C**) Proximal colonic muscle strip. (**B**,**D**) Summarized bar graph analyzing tension recording by vehicle (gray), 1 μM (pink), and 10 μM (salmon) LUB treatment. (**B**) Vehicle (*n* = 8), 1 μM (*n* = 4), and 10 μM (*n* = 8). (**D**) Vehicle (*n* = 13), 1 μM (*n* = 5), and 10 μM (*n* = 8). n.s: non-significant, and *** *p* < 0.001.

Therefore, expecting that LUB would differentially contribute to motility across specific segments of the colon, the contractile wave induced by LUB treatment in the distal colonic muscular layer was measured. The motility of the proximal colon clearly showed alterations in the regularity of the contractile wave pattern (Figure 2A), yet the the area under the curve (AUC) demonstrated no significant variance compared to that before LUB treatment (72.90 ± 10.72% of 1 µM, 95.51 ± 11.17% of 10 µM) (Figure 2B). There were no differences in the changes in baseline or wave amplitude (2.32 ± 0.17 g of vehicle, 2.16 ± 0.13 g of 1 µM and 2.34 ± 0.09 g of 10 µM). Although the pattern of the waveforms clearly exhibited alterations, there was no difference in overall frequency (5.28 ± 0.47 cpm of vehicle, 4.33 ± 0.67 cpm of 1 µM and 5.29 ± 0.42 cpm of 10 µM). However, in the distal colonic muscle strip, treatment with 10 µM LUB evoked enhanced motility (70.50 ± 8.26% of 1 µM, 223.20 ± 23.27% of 10 µM) (Figure 1C,D). Treatment with 1 µM LUB significantly enhanced motility in the distal colon, inducing a modest increase in wave frequency (2.92 ± 0.35 cpm of vehicle, 3.60 ± 0.24 cpm of 1 µM and 5.13 ± 0.44 cpm of 10 µM) and a particularly significant increase in amplitude (0.64 ± 0.10 g of vehicle, 0.90 ± 0.19 g of 1 µM and 3.73 ± 0.28 g of 10 µM).

In summary, CLC-2 expression was absent in the muscular layer, suggesting LUB’s effects on motility are independent of direct muscle layer mechanisms. While LUB treatment did not significantly alter baseline motility or frequency in the proximal colon, it markedly increased motility, particularly amplitude, in the distal colon at higher concentrations, indicating a differential impact on colonic segments.

### 2.2. Lubiprostone Increases Colonic Contraction Depending on the Activation of Prostaglandin E2 Receptors

**Figure 3:**
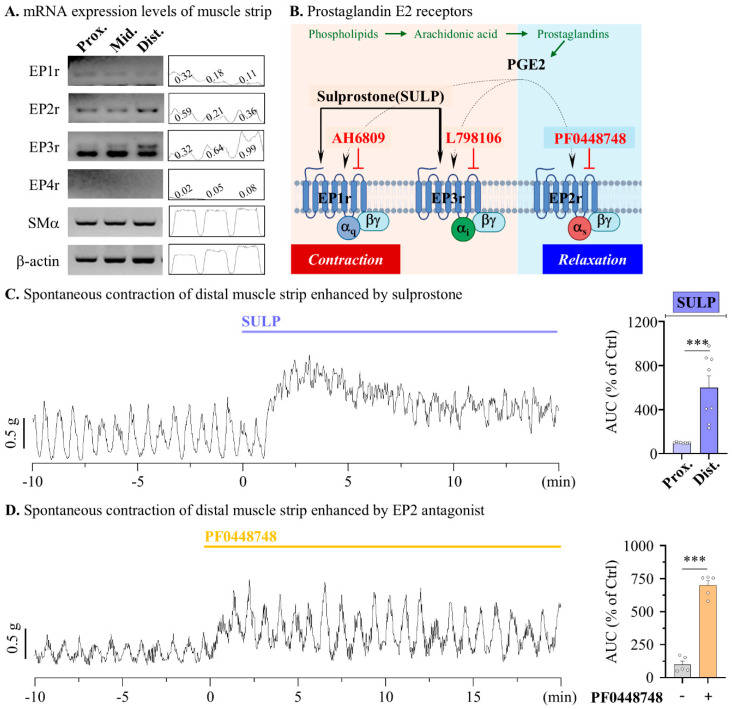
Modulation of contractile responses through EP receptor activation in mouse colonic muscle strips. The activation of EP3 receptor enhances distal colonic contraction in mouse. (**A**) Representative blot (left) of reverse transcription PCR in muscle layer of mouse colon and densitometer (right) analysis relative to β-actin. (**B**) The summarized scheme of muscle contraction related to EP receptor activation and agonists (arrow) and antagonists (blunt arrow) of EP receptors. (**C**,**D**) Summary bar graph analysis of tension recording by 1 μM SULP (blue), vehicle (gray), and 1 μM PF0448748 (yellow) treatments. (**C**) *n* = 6. (**D**) *n* = 5. *** *p* < 0.001.

Therefore, the next aim was to identify the physiological mechanisms underlying the enhancement of LUB-induced colonic motility, excluding CLC-2 activation. Previous reports have suggested that LUB, a derivative of prostaglandin, contributes to the activation of EP receptors, and the stimulation of EP receptors evokes an increase in smooth muscle contraction [27,28]. To confirm the contribution of EP receptors to the colonic muscular layer, we first isolated the muscle layer from the proximal, middle, and distal colons using RT-PCR to measure the RNA expression levels of the EP receptor subtypes (Figure 3A). For EP1 and EP2, no significant differences in expression were observed from the proximal to the distal colon, and EP4 expression was not detected in the mouse colonic muscle layer as a reference by β-actin. However, EP3 expression gradually increased from the proximal to the distal colon. Therefore, to determine whether the expression of EP receptors contributed to each colonic segment (Figure 3B), the contractile wave was assessed after ligand treatment. Specifically, 10 μM PGE2, an activator of all EP receptor subtypes, was applied to the muscular strip for this measurement. Proximal colonic spontaneous contractions showed a reduction in the muscle contractile wave by more than 80% following PGE2 treatment (Figure S1A). Conversely, a twofold increase in the muscle contractile wave was observed in the distal colonic spontaneous contractions (Figure S1B). To evaluate the regulatory effect of EP receptor activation on colonic motility, spontaneous contractions induced by specific agonist and antagonist treatments for each subtype were measured. Inhibition of the Gs pathway associated with EP2 evoked a contractile increase in both the frequency and amplitude of the distal muscle. (522.60 ± 45.48% in 1 μM GW62738X) (Figure 3D).

**Figure 4:**
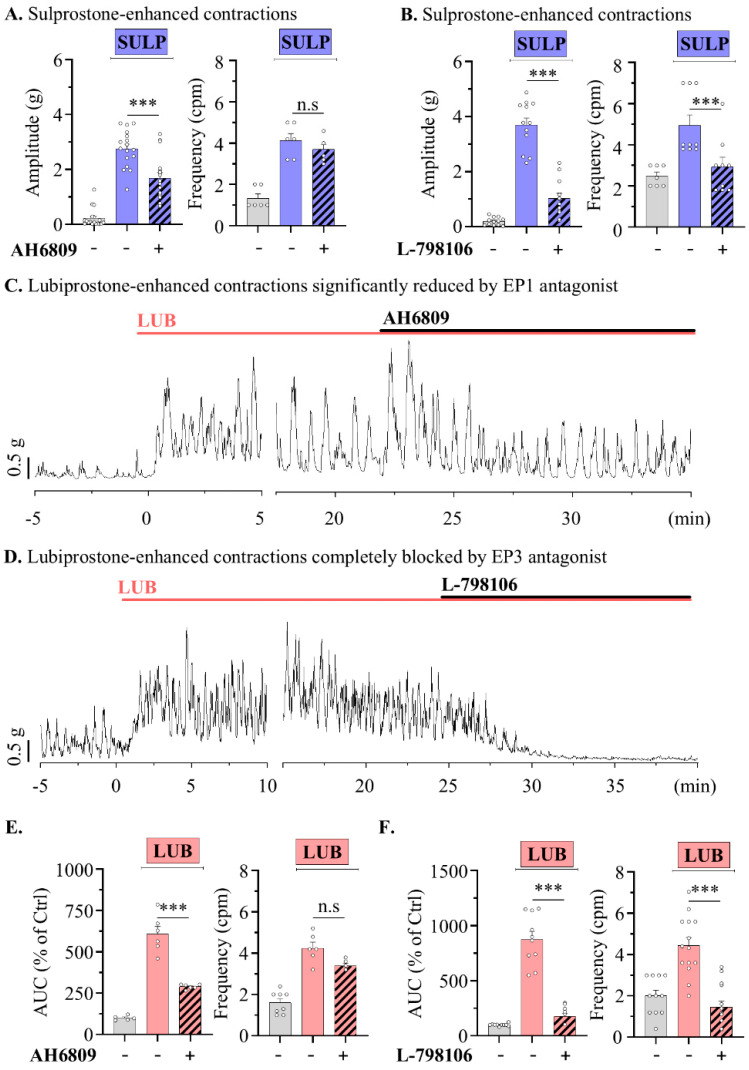
Lubiprostone-enhanced peristalsis wave of distal colonic muscle is attenuated by EP3 antagonist. (**A**,**B**) Summary bar graph analysis of tension recording by vehicle (gray), 1 μM SULP (blue), and 1 μM antagonist (striped pattern) treatments. (**A**) *n* = 6. (**B**) *n* = 5. (**C**,**D**) Representative traces of muscle strips in mouse distal colon. (**E**,**F**) Summary bar graph analysis of tension recording by vehicle (gray), 1 μM LUB (pink), and 1 μM antagonist (striped pattern) treatments. (**E**) *n* = 9. (**F**) *n* = 13. n.s: non-significant, and *** *p* < 0.001.

In contrast, treatment with sulprostone (SULP), an agonist for EP1 and EP3 receptors, increased tonic contractions of the distal muscle strip, accompanied by an elevation in the contractile baseline (599.20 ± 105.90%) (Figure 4A). A previous report showed that EP3 activation contributed significantly to the enhancement of middle colonic contractions compared to EP1 activation in rats [28]. To clearly distinguish the contributions of EP1 and EP3 in modulating colonic motility, SULP-induced contractile waves were measured following treatment with specific antagonists in the distal segments. The treatment of 1 µM AH6809, an antagonist for EP1, EP3, and mildly EP2, reduced the tonic contractions induced by SULP, decreasing from 607.62 ± 46.79% to 287.32 ± 8.12%; however, the peristaltic frequency was not significantly altered (Figure 4A). Meanwhile, 1 µM of L-798106, an EP3 antagonist, completely reduced the induced tonic and peristaltic contractions from 876.11 ± 72.25% to 177.2 ± 26.20% and also significantly decreased the frequency of contractions (4.93 ± 0.52 to 2.93 ± 0.46 cpm) (Figure 4B). This suggests that the EP3 receptor significantly contributes to the motility of the distal colon. Also, to determine whether EP1 and EP3 contribute to the increase in intestinal motility induced by LUB, the effects of 1 µM AH6809 and L-798106 treatments on LUB-induced activity were examined. The AH6809 treatment reduced the contractile wave to half its level (294.78 ± 23.74% to 150.06 ± 11.32%), but the frequency of contractions remained unchanged (Figure 4C,E). As expected, the treatment with L-798106 completely reduced the colonic spontaneous contraction induced by LUB (268.62 ± 14.22% to 22.81 ± 5.00%) and also significantly decreased the frequency of contractions (4.44 ± 0.39 to 1.46 ± 0.28 cpm) (Figure 4D,F).

As a result, we investigated the impact of E-series prostanoid (EP) receptors on colonic motility, revealing that while EP1 and EP2 receptors showed consistent expression throughout the colon, EP3 expression increased towards the distal end. Functional tests demonstrated that the activation of EP receptors by PGE2 differently affected motility along the colon, enhancing contractions significantly in the distal part. The treatments with specific antagonists such as AH6809 and L-798106 further highlight EP3’s dominant role in modulating colonic motility, particularly in enhancing responses to the prostaglandin derivative LUB.

### 2.3. Lubiprostone-Induced Colonic Contraction via EP Receptor Modulation Is Dependent on TRPC4 Activity in Mouse Colonic Myocytes

**Figure 5:**
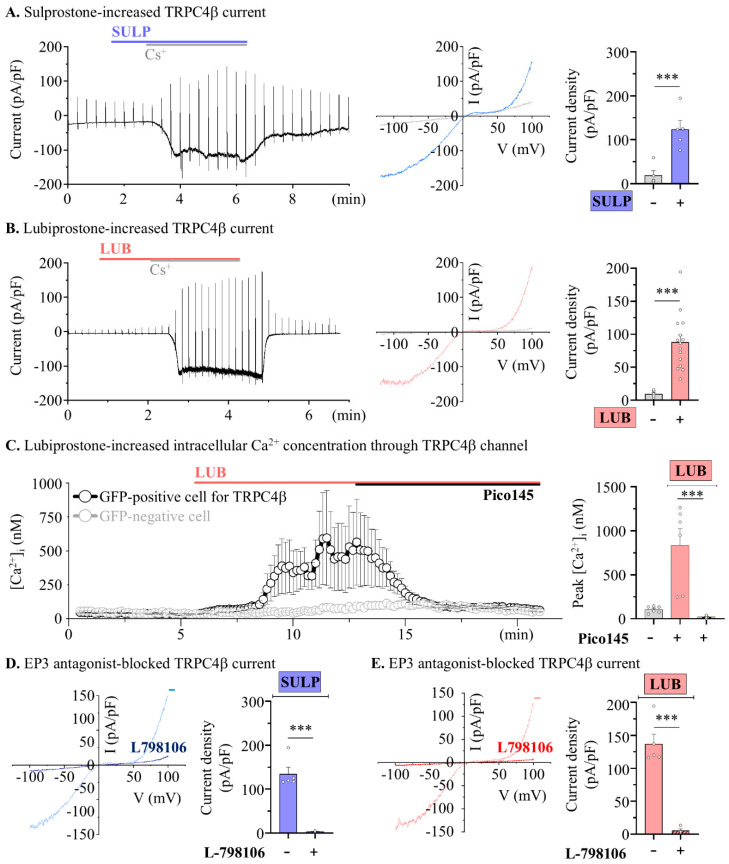
Lubiprostone increases TRPC4β activity by stimulating EP3 receptor. (**A**) The representative blot (left) and I-V curve (right) of whole-cell current in TRPC4β-overexpressed cells. (**B**) Summary box and whisker plot analysis of current density (pA/pF). (**C**) The summarized traces of intracellular calcium concentration (left) and summary box and whisker plot of 10 μM LUB and 10 nM Pico145 treatments in TRPC4-positive (black) and -negative cells (gray) (*n* = 6). (**D**,**E**) Representative I-V curve (left) and summary box and whisker plot (right) analysis of TRPC4 current density. Co-treatments of 1 μM SULP (blue) and 1 μM L-798106 (striped pattern) (*n* = 6). (**E**) Co-treatments of 10 μM LUB (red) and 1 μM L-798106 (striped pattern) (*n* = 6). *** *p* < 0.001.

Our previous reports suggest that stimulation of opioid receptors, which activate the Gi-pathway [23], enhances colonic motility through activation of the TRPC4 channel [24]. Therefore, the TRPC4 channel was used as a target protein for colonic contractions mediated by EP3 receptor activation. Measurement of RNA expression in HEK293 cells confirmed the endogenous expression of EP2, EP3, and EP4 [29]. Our reverse transcription PCR results were similar for HEK293 cells (Figure S2). Subsequently, to investigate TRPC4 activation evoked by the EP3 receptor, whole-cell current was measured in TRPC4β-overexpressed HEK293 cells following SULP treatment. The TRPC4β current increased from 19.32 ± 10.28 pA/pF in the vehicle to 123.00 ± 19.20 pA/pF following SULP treatment (Figure 5A). Furthermore, TRPC4β current was increased from 9.37 ± 2.01 pA/pF to 88.29 ± 10.77 pA/pF via 10 μM LUB treatment (Figure 5B). TRPC4β current was enhanced by EP3 stimulation, with the endogenous level of EP3 expression in the HEK293 cells being sufficient for TRPC4β activation. Next, we confirmed that the intracellular calcium concentration was increased by the TRPC4 channel, which is vital for smooth muscle contraction. TRPC4β-GFP was overexpressed in HEK293 cells, and the intracellular calcium levels were measured in both GFP-positive and -negative cells through the analysis of Fura-2 intensity. In the TRPC4β-positive cells, the basal Ca^2+^ level of 105.23 ± 15.35 nM significantly increased to 835.50 ± 189.22 nM with the LUB treatment; in contrast, the Pico145 treatment, a selective TRPC4 blocker, significantly decreased the calcium concentration to 22.94 ± 5.06 nM (Figure 5C). Moreover, SULP-/LUB-evoked TRPC4 currents were significantly diminished by the EP3-selective blocker, L-798106 (Figure 5D,E). SULP-evoked TRPC4 current was decreased by the 1 µM L-798106 co-treatment (from 137.12 ± 19.18 pA/pF to 3.15 ± 0.37 pA/pF), and LUB-evoked TRPC current was also decreased by the 1 µM L-798106 co-treatment (from 137.12 ± 14.86 pA/pF to 5.65 ± 2.66 pA/pF). Consequently, LUB can indirectly activate TRPC4 through EP3 receptor modulation, inducing an increase in the intracellular Ca^2+^ concentration.

**Figure 6:**
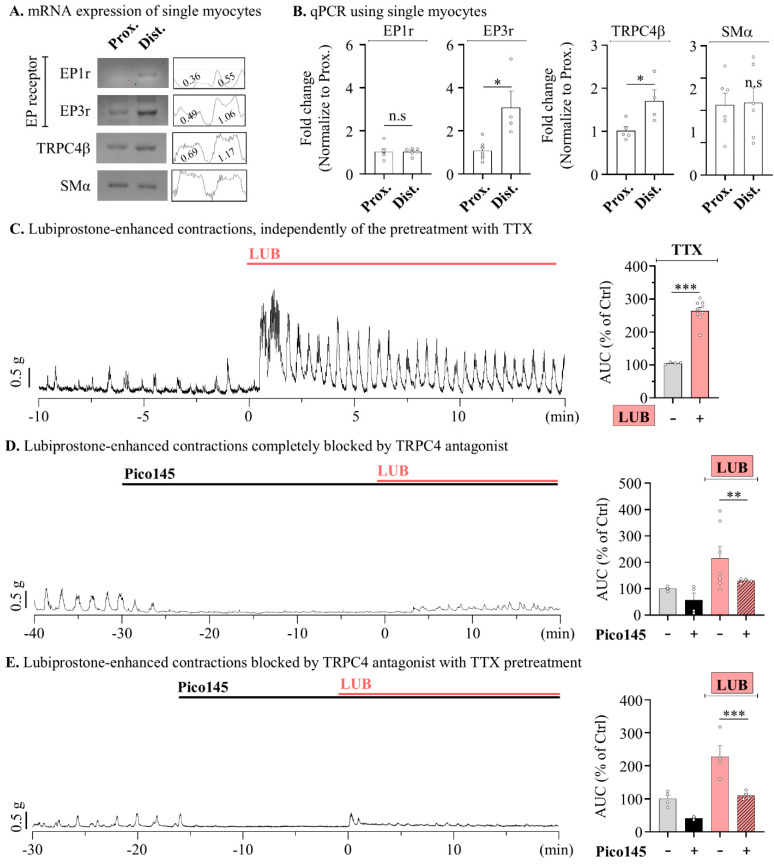
Lubiprostone-evoked peristalsis wave is inhibited thought TRPC4 activation in mouse colonic myocytes. (**A**) Reverse transcription PCR representative blot (left) of selected single myocytes in the mouse colon and densitometer (right) analysis relative to β-actin. (**B**) Summary box and whisker plot analysis of RT-qPCR of selected single myocytes. (**C**–**E**) Representative traces of contractile wave (left) and summary box and whisker plots (right) analyzing the AUC of tension recording. Cotreatments of 10 nM Pico145 (black), 10 μM LUB (red) and co-treatment (striped pattern) (**D**,**E**) of 1 μM TTX for 10 min. (**C**) *n* = 7. (**D**) *n* = 7. (**E**) *n* = 4. n.s: non-significant, * *p* < 0.05, ** *p* < 0.01 and *** *p* < 0.001.

To further distinguish the physiological contributions of contractile sources, LUB-evoked colonic motility was measured under conditions that ruled out each factor. So, we aimed to identify the main source of the contraction mechanism within myocytes; thus, colonic myocytes were isolated through single-cell picking harvesting with the patch clamp technique [30], and expression levels were verified via RT-PCR. When investigating the expression of EP1 and EP3 receptors, presumed target proteins of LUB, both were found to be more highly expressed in the distal colonic myocytes, with the expression of EP3 significantly elevated in the distal myocytes compared to that in the proximal myocytes (Figure 6A). In contrast, real-time PCR analysis showed almost no change in EP1 expression, whereas EP3 expression was approximately threefold higher in the distal myocytes than in the proximal myocytes (Figure 6B). Additionally, the expression of TRPC4 was also observed to be higher in the distal myocytes than in the proximal myocytes, suggesting a potential for sensitive contractile responses to LUB-induced EP3 stimulation.

Additionally, given that our measurements were obtained from colonic muscle strips isolated from the mucosal layer, central nervous system signaling could be excluded. However, the ENS regulatory mechanism still contributed. Therefore, to completely rule-out ENS stimulation, 1 μM tetrodotoxin (TTX) was used to inhibit neural activity, and spontaneous contractions induced by LUB were observed. Similar to the results shown in Figure 1 and Figure 2, the pre-treatment with TTX still resulted in a significant increase in spontaneous contraction of the mouse distal colonic muscle layer by 263.78 ± 11.12% upon LUB treatment (Figure 6C). LUB-induced contractions were increased despite neuronal inhibition being ruled out by TTX, suggesting an independent mechanism from the ENS. Finally, to investigate whether TRPC4 activity contributes to the contractile wave induced by LUB, the LUB-evoked contractile wave was measured with Pico145 pre-treatment. Initially, the treatment with Pico145 significantly reduced the basal level of spontaneous contractions by more than 50%, and the co-treatment with LUB resulted in only a 130.13 ± 4.19% increase, contrasting with the more than twofold increase previously observed in tonic contractions (Figure 6D). Similarly, the increase to 228.34 ± 32.95% observed upon LUB treatment in the presence of TTX was also reduced to 110.32 ± 6.45% with the Pico145 co-treatment (Figure 6E).

Consequently, our results suggest a mechanism of LUB-induced colonic motility, demonstrating that contractions independently evoke the ENS and are primarily mediated by EP3-receptor-stimulated TRPC4 channel activation. TRPC4 activation induced a significant increase in the intracellular Ca^2+^ concentration and membrane potential. The upregulation of EP3 and TRPC4 in distal colonic myocytes compared to that in proximal myocytes suggests segmental specificity. Moreover, the reduction in LUB-evoked contractions by TRPC4 blockers establishes the critical role of TRPC4 in mediating colonic contractions. Thus, these findings not only propose the EP3 receptor as a therapeutic target beyond CLC-2 activation by LUB but also highlight the importance of TRPC4 channel interactions in enhancing colonic motility.

## 3. Discussion

In summarizing our study on the effects of LUB on colonic motility, it contributed to smooth muscle contractions in mouse distal colonic muscle strips, and our findings were grouped into three observations: (1) Independent of previous expectations of CLC-2 chloride channel activation in the GI mucosal layer, our results suggest that the activation of EP3 receptors by LUB is the primary pathway driving smooth muscle contractions. These results were supported by the increase in tonic and phasic contractions observed in distal colonic muscle strips treated with LUB, which was induced by the activation of TRPC4. (2) The differential expression of EP receptors in various colonic segments and their gradational increase from the proximal to the distal colon explains the segment-specific enhancement of colonic motility in response to LUB treatment. (3) Additionally, TRPC4 channels facilitated the contractile response to LUB-induced EP3 activation, highlighting their role in enhancing colonic motility through Gi-pathway signaling. Through these observations, our study not only broadens the understanding of the therapeutic mechanism of LUB but also indicates potential pathways for developing targeted therapies, such as TRPC4 activation, for constipation and related GI motility disorders.

As previously demonstrated, the efficacy of LUB in enhancing colonic motility can be attributed to its action beyond the previously understood mechanism of CLC-2 activation. The significant increase in the peristalsis wave following LUB treatment in the distal colonic muscular strips, despite the absence of CLC-2, suggests an alternative pathway through the EP3 receptor. This observation prompted further investigation into the role of TRPC4 channels in mediating the LUB-induced enhancement of colonic contraction. In particular, increased contractility in the distal colon can be inferred from the anatomical and physiological requirements of the GI tract [31]. The proximal colon, which is primarily involved in water and electrolyte absorption, exhibits lower basal spontaneous contraction frequencies and higher contraction amplitudes. In contrast, the distal colon, which is important for fecal storage and evacuation, displays higher frequencies of basal spontaneous contractions but lower amplitudes. LUB treatment boosts the contractile wave in the distal segment, underlining the therapeutic necessity of segment-specific interventions to effectively augment motility. The presence of sphincters and valves at the terminal portions of the GI tract suggests the necessity for enhanced contractility, especially in the colon, where stool fluidity diminishes and solidity increases, necessitating greater contraction forces for effective fecal propulsion [25]. Consequently, activation of the TRPC4 channel implies a heightened sensitivity to the mechanisms of mass motility. Our findings strongly support the idea that the LUB modulation of contraction strength in the distal colon aligns with the physiological demand for higher contractile forces in this segment, enhancing bowel evacuation. Our findings provide a novel perspective on the mechanism by which LUB enhances colonic motility, particularly through its action on EP3 receptors and TRPC4 channels. When compared to other prokinetic agents, such as prucalopride (5-HT4 receptor agonist) and linaclotide (guanylate cyclase-C agonist), LUB demonstrates a unique, segment-specific effect in the distal colon, which is not typically observed with these other agents. Prucalopride, for example, increases colonic motility by enhancing serotonin release from enteric neurons, and linaclotide works primarily by increasing fluid secretion into the intestinal lumen. This highlights LUB’s potential as a therapeutic agent specifically targeting motility in the distal colon, where stool storage and propulsion require stronger contractile forces.

Moreover, the pharmacokinetics of LUB, similar to those of prostaglandins, are defined by its metabolism by carbonic anhydrase in the liver, which is characterized by a rapid half-life and excretion. Unlike the general-purpose laxative prucalopride (5-HT4), which has a half-life of approximately 24 h, LUB has a relatively short half-life of from 0.9 to 1.4 h [32]. This rapid metabolism in the liver, along with a systemic absorption rate of <10%, determines its pharmacokinetic characteristics [33,34]. Furthermore, as an oral therapy, LUB has a pharmacokinetic profile that allows its effects to begin immediately upon exposure to the GI tract without the need for metabolic processing [35,36]. Typically, LUB is prescribed at doses ranging from 24 to 48 μg per day. While the concentrations used in our results, ranging from 1 to 10 μM, are comparatively high, the local concentrations at the site of action may indeed be higher. Consequently, identifying the specific contractile segment of LUB is important for determining its usage and dosage.

Therefore, unlike the proximal colon, where constipation is improved through osmotic pressure, LUB directly contributes to intestinal contraction in the distal colon, indicating direct therapeutic efficiency. Therefore, the differential expression of EP receptors along the colonic muscle layer and their specific contribution to the regulation of colonic motility are key suggestions of our study. This segment-specific effect can be attributed to the distinct expression patterns of EP receptors across the entire colon, with EP3 receptors showing a gradual increase in expression from the proximal to the distal colon. Our findings demonstrate that while EP2 and EP4 receptor activation induces intestinal relaxation, the activation of EP1 and EP3 receptors, particularly EP3, significantly contributes to the enhancement of colonic motility in the distal colon. This segment-specific modulation of colonic motility by EP receptors explains the mechanism of LUB in constipation treatment, especially considering the mechanisms of fecal storage and propulsion in the distal colon. Furthermore, the involvement of TRPC4 channels as downstream effectors in the EP-receptor-mediated pathway defines the therapeutic mechanism of LUB, with indirect stimulation of TRPC4 channels inducing calcium ion influx, which is essential for smooth muscle contraction [24]. Although our experiments were confined to distinguishing sections of the murine colon, the identification of TRPC4 as a target protein involved in mass motility suggests a potential therapeutic mechanism for patients with constipation.

In contrast, agents such as misoprostol, an EP1/EP3 agonist often used for its effects on gastric and uterine smooth muscle, have not been as extensively studied for their direct effects on TRPC4 channels. While misoprostol is known to increase motility in other regions of the gastrointestinal tract, its specific interactions with TRPC4 in colonic smooth muscle remain unclear [37]. Our data suggest that targeting the TRPC4 channel through EP3 receptor activation may offer a more focused approach for modulating colonic motility, particularly in conditions such as slow transit constipation. Additionally, research on EP2 and EP4 receptor agonists like butaprost and CAY10580, respectively, has shown that these receptors are more commonly associated with smooth muscle relaxation rather than contraction [38]. In contrast to our findings with LUB and TRPC4 activation, the activation of EP2 and EP4 typically leads to decreased motility by increasing cAMP levels, which results in smooth muscle relaxation. This demonstrates the importance of selectively targeting EP3 and TRPC4 for enhancing colonic motility while avoiding the counteractive effects associated with EP2 and EP4 receptor activation.

Meanwhile, LUB treatment did not significantly increase the contractile force within the proximal colonic muscle layer, modifying the regularization of contraction waveforms (Figure 2A). This observation indicates that LUB may modulate the contractile complex in the proximal colon. The underlying mechanism responsible for the modulation of the contraction patterns, which was not investigated in the present study, may involve multiple physiological processes. The observed regularization could be attributed to the influence of LUB on ion channel functionality or signal transduction pathways involved in regulating myogenic rhythmicity. Additionally, the potential of LUB to modulate the activity of ICCs, integral to synchronizing smooth muscle contractions, may have contributed to altered waveform dynamics [12]. Additionally, LUB treatment may indirectly modify other endogenous regulators of contractile wave patterns. However, our investigation did not extend to a direct mechanism of LUB rhythmic regulation beyond the function of CLC-2 or ICC and neuromodulator signaling in the proximal colonic muscle layer.

The partial mitigation of LUB-induced colonic contractions through TRPC4 inhibition, as evidenced by our results, implies the existence of additional modulatory pathways activated by LUB. Although the application of Pico145, a selective TRPC4 inhibitor, significantly reduced the enhanced contractile response, this effect was not entirely negated (Figure 6C–E). This partial reduction suggests that TRPC4 channels constitute a primary but not exclusive route through which LUB exerts its pharmacological effects on colonic motility. Nevertheless, the predominance of TRPC4 in response to LUB is evident, as its inhibition markedly diminished, although it did not completely eliminate, LUB-induced contractions. This suggests that TRPC4 contributes to the physiological mechanisms of LUB and highlights its potential as a therapeutic target. However, a comprehensive understanding of the LUB mechanisms requires the investigation of additional contributors to colonic motility.

Furthermore, this study suggests that selective targeting of EP3 receptors and TRPC4 channels could provide more effective treatment options for patients with slow transit constipation (STC) by enhancing contractility specifically in the distal colon. Clinically, the combination of LUB with other prokinetic agents could offer a synergistic approach, improving overall gastrointestinal motility while minimizing side effects such as excessive fluid secretion or generalized hypermotility, which are commonly associated with non-segment-specific agents like linaclotide or prucalopride. This suggests a new strategy for combination therapies aimed at treating constipation with a more targeted and personalized approach.

In conclusion, our study advances our understanding of pharmacological therapies for modulating colonic motility. By discovering the effective location of colonic segments for LUB to enhance muscle motility, our report offers therapeutic insights into the structural and functional differentiation of the colon. These findings not only redefine our understanding of the pharmacological action of LUB but also underscore the therapeutic relevance of targeting EP3 receptors and TRPC4 channels. By demonstrating the significant modulation of contraction patterns across different colonic segments, particularly enhanced contractility in the distal colon, which is essential for effective bowel movements, our study points towards new directions for tailored therapeutic approaches to treat constipation and other related FGIDs.

## 4. Method

### 4.1. Cell Culture, Transient Transfection, and Chemicals

HEK293 cells were cultured in Dulbecco’s Modified Eagle Medium (DMEM) obtained from Welgene (Gyeongsan, Republic of Korea), supplemented with 10% fetal bovine serum and 1% penicillin–streptomycin mixture. The cells were housed in a controlled environment at 37 °C with a humidified atmosphere of 5% CO_2_. For transient transfection, cells were seeded at a density appropriate for achieving 70–80% confluence by the time of transfection. Transfection was carried out using the X-tremeGENE9 DNA Transfection Reagent (Sigma-Aldrich, St. Louis, MO, USA), following the supplier’s instructions; typically, 500 ng of plasmid was mixed with the reagent in serum-free medium and added to the cells. Cells were further incubated for 24 to 48 h before assays were conducted to evaluate gene expression and protein functionality. All additional chemicals, including reagents for cell maintenance and assays, were procured from Sigma-Aldrich, ensuring high quality and consistency across all experimental protocols.

### 4.2. Mechanical Tension Recordings of Mouse Colonic Muscle Layer

Isolation and preparation of the colonic muscle layer were conducted on C57BL/6N mice aged between 30 and 60 days, without distinction of sex. These procedures were performed with full approval from the ethics committee of Chosun University and in strict adherence to the National Institutes of Health guidelines for the care and use of laboratory animals under approval number CIACUC 2024-S0036, following the ethical standards and regulations set by the Chosun University IACUC.

Following exposure to isoprene and subsequent sacrifice, approximately 1.5 cm just after the cecum was designated as the proximal colon, and 1 cm from the rectum was identified as the distal colon. To analyze the contractile wave, colonic muscle strips with the mucosal layer gently removed using forceps were incised parallel to the circular muscle layer. The mucosal and submucosal layers were removed in a Ca^2+^-free Hanks’ solution containing (in mM) 135 NaCl, 5 KCl, 5 glucose, and 5 HEPES, with the pH adjusted to 7.4 using NaOH. These muscle strips were subsequently attached to an isometric force transducer (Harvard Apparatus, Holliston, MA, USA) and immersed in a 30 mL organ bath. The bath contained bicarbonate solution, prewarmed to 36.5 ± 0.5 °C and preoxygenated. A 60 min stabilization period was followed by an equilibration period under a resting force of 1 g to prepare the muscle strips for recording. All procedures were conducted in preoxygenated Krebs–Ringer bicarbonate solution. The composition of this solution included the following (in mM): 120.4 NaCl, 15.5 NaHCO_3_, 5.9 KCl, 11.5 glucose, 1.2 NaH_2_PO_4_, 1.8 CaCl_2_, and 1.0 MgCl_2_. The pH of the solution was maintained at 7.4 at a temperature of 37 °C, with a gas mix of 97% O_2_ and 3% CO_2_ to ensure proper oxygenation.

The analysis focused on AUC over 5 min for the contractile wave before and after drug application. Mechanical responses were captured and digitized using the AcqKnowledge software (Harvard apparatus), and offline analysis was conducted using Clampfit (version 10.7, Molecular Devices, San Jose, CA, USA). In some experiments, to exclude neural contributions to the responses induced by the circular muscle strip, TTX was administered 10 min prior to chemical application.

### 4.3. Electrophysiology for Recording the TRPC4 Current

For electrophysiological assessment of TRPC4 currents, recordings were made at ambient room temperature using borosilicate glass capillaries. Capillary pipettes were pulled to achieve resistances of 3–4 MΩ using a PC-10 puller. The currents were measured using an Axopatch 200B amplifier (Molecular Devices, San Jose, CA, USA) and Digidata 1550B Interface (Molecular Devices, San Jose, CA, USA), and the data were subsequently analyzed using the Origin 2022b software (OriginLab Co., Northampton, MA, USA). The intracellular pipette solution for whole-cell recordings consisted of the following (in mM): 140 CsCl, 10 HEPES, 0.2 Tris-GTP, 0.5 EGTA, and 3 Mg-ATP. The pH was adjusted to 7.3 using CsOH. The extracellular bath solution, or normal Tyrode’s solution, contained (in mM) 135 NaCl, 5 KCl, 2 CaCl_2_, 1 MgCl_2_, 10 glucose, and 10 HEPES, with the pH balanced to 7.4 with NaOH. A Cs^+^-rich external solution was prepared by substituting the monovalent cations (NaCl and KCl) in normal Tyrode’s solution with an equivalent concentration of CsCl. Voltage ramp pulses ranging from +100 to −100 mV over a period of 0.5 s were applied at 10 s intervals, with the holding potential maintained at −60 mV. The corresponding current (I)–voltage (V) relationship is depicted using Roman numerals in the current trace graph. Data representation for inward current amplitudes at −60 mV utilized box and whisker plot summaries.

### 4.4. Calcium Imaging

Ratiometric measurements of [Ca^2+^]*_i_* were performed using Fura-2-AM (Molecular Probes, Eugene, OR, USA). The cells were suspended and loaded with 2 μM of Fura-2-AM for 30 min at 37 °C. Fura-2 fluorescence was measured at 510 nm emission with 340/380 nm dual excitation using a pE-340 Fura illuminator (CoolLED, Andover, UK). The experiments were performed using normal Tyrod’s solution. All image analyses were performed using the NIS-Elements imaging software Ar 5.20 (Nikon, Tokyo, Japan).

### 4.5. Myocyte Harvest and Complementary DNA Synthesis

Strips of colonic muscle were then transferred to the same solution containing 0.1% collagenase (Worthington Biochemical Co., Lakewood, NJ, USA), 0.2% bovine serum albumin (Sigma), 0.1% trypsin inhibitor (Sigma), and 0.01% papain (Sigma). The tissues were incubated in the enzyme solution at 37 °C for 10–15 min, followed by washing with Ca^2+^-free Hanks’ solution. Single cells were obtained by gentle agitation using a wide-bore glass pipette. Isolated cells were maintained at 4 °C until use. Subsequently, a patch lamp apparatus was employed for the single-cell selection of myocytes, which were harvested in normal Tyrode’s solution. Total RNA was extracted from the mouse colonic mucosa/muscle layer or isolated from colonic myocytes using TRIzol Reagent (Invitrogen, Carlsbad, CA, USA) according to the manufacturer’s instructions. RNA integrity was verified by electrophoresis on a 1–2% agarose gel. Complementary DNA (cDNA) was synthesized from 1 µg of total RNA using the PrimeScript 1st strand cDNA Synthesis Kit (TaKaRa, Kusatsu, Japan) following the manufacturer’s protocol.

### 4.6. Reverse Transcription Polymerase Chain Reaction

PCR amplification was conducted using 2 µL of the cDNA (200 ng/µL) template in a 50 µL reaction volume with the specific primers and β-actin. The PCR reaction mix included 50 µL of 0.25 µL Ex taq polymerase (TaKaRa), 2 µL of each primer (10 pM), 4 µL of dNTP (2.5 mM), 5 µL of 10× Ex Taq buffer, and 6 µL of nuclease-free water. The amplification conditions were as follows: initial denaturation at 95 °C for 3 min, followed by 35 cycles of denaturation at 98 °C for 10 s, annealing at 56 °C for 30 s, and extension at 72 °C for 45 s, with a final extension at 72 °C for 5 min. The PCR products were analyzed by electrophoresis on a 2% agarose gel stained with blue mango (BioD, Incheon, Republic of Korea) and visualized under UV light.

### 4.7. Real-Time Polymerase Chain Reaction

Quantitative PCR was performed using the TOPreal™ SYBR^®^ Green Master Mix (Bio-rad, Hercules, CA, USA) on a CFX96 Real-Time PCR Machine (Bio-rad). The reaction mixture of 20 µL contained 10 µL of 2× SYBR^®^ Green Master Mix, 1 µL of each primer (10 µM), 2 µL of cDNA template, and 6 µL of nuclease-free water. The thermal cycling conditions included an initial uracil-DNA glycosylase activation at 50 °C for 2 min, polymerase activation at 95 °C for 2 min, followed by 40 cycles of denaturation at 95 °C for 15 s and annealing/extension at 56 °C for 1 min. The specificity of the PCR products was confirmed using melting curve analysis. Relative gene expression levels were calculated using the 2^(−Δ∆Ct)^ method, with β-actin serving as the normalization control. The primer sequences used for reverse transcription (RT)-PCR and reverse transcription quantitative real-time PCR are shown in Supplementary Table S1.

### 4.8. Statistics

All statistical analyses were conducted using either the Origin 2022b or Prism 10 (GraphPad) software. All data are presented using box and whisker plots, with whiskers denoting the range according to Tukey’s test and dots marking outliers. For comparisons between two groups, Student’s *t*-test was applied. For multiple group comparisons, one-way analysis of variance (ANOVA) was used, followed by post hoc tests (Tukey’s multiple comparison test) to identify specific group differences. Assumptions for normality and homogeneity of variance were tested using the Shapiro–Wilk test and Levene’s test, respectively. If these assumptions were violated, non-parametric tests were applied accordingly. The significance levels are indicated as follows: n.s. for non-significant, * *p* < 0.05, ** *p* < 0.01, and *** *p* < 0.001 when compared to the control group.

## Data Availability

The datasets analyzed during the study are available from the corresponding author on reasonable request.

## Supplementary Materials

The following supporting information can be downloaded at: https://www.mdpi.com/article/10.3390/ph17101327/s1, Table S1: The primer sequence for PCRs. Figure S1: EP receptors level in HEK293 cell. Figure S2: The contractile wave induced by EP antagonists.
