# Supplementary material for: Lubiprostone Improves Distal Segment-Specific Colonic Contractions through TRPC4 Activation Stimulated by EP3 Prostanoid Receptor
Source: Pharmaceuticals (Basel). 2024 Oct 4;17(10):1327. doi: 10.3390/ph17101327 (PMC11509986; doi:10.3390/ph17101327)
Supplement: Supplementary file 1 [file pharmaceuticals-17-01327-s001.zip › pharmaceuticals-3217365-supplementary.pdf]

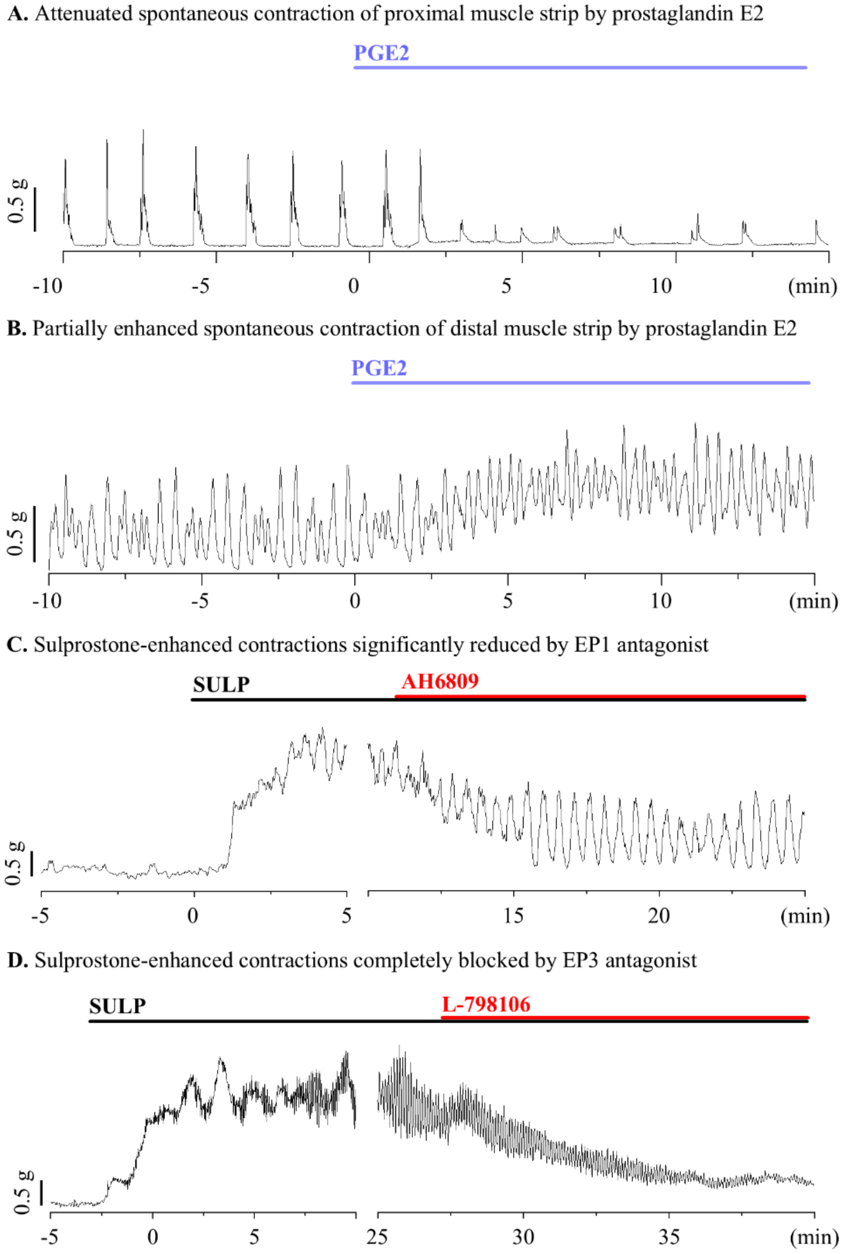

Figure S1. A-D. The representative traces of contractile wave.

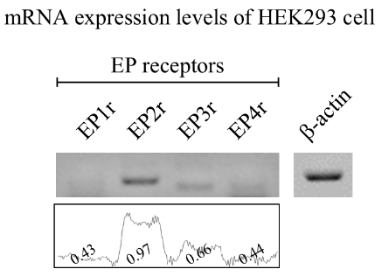

Figure S2. The representative blot of reverse transcript PCR in HEK293 cell (*top*) and densitometer (*bottom*) analyzed relative to β-actin.

Table S1. The primer sequence used in reverse-transcription PCR and quantitative real-time PCR.

Target RNA		PCR primer sequence	Size (bp)
EP1	Forward	5'-TCATGGTGGTGTCGTGCATC-3'	185
	Reverse	5'-AAGCAGTTGGCGCAGCA-3'	
EP2	Forward	5'-GAGGAGACGGACCACCTCAT-3'	177
	Reverse	5'-GGCAAAGACCCAAGGGTCAA-3'	
EP3	Forward	5'-CACTGGTATGCCAGCCACAT-3'	149
	Reverse	5'-GTGCTGATGAAGCACCACGT-3'	
EP4	Forward	5'-GTCCAGTCGATGAAGCACCAG-3'	174
	Reverse	5'-CTACCTGGCCATCAACCATGC-3'	
TRPC4	Forward	5'-AAGAAATTACTCGTCAACAGGC-3'	209
	Reverse	5'-GTAAACCCAAAGTGTCCGTATT-3'	
CLC-2	Forward	5'-CCTTCCAGCTGGTGGAGC-3'	218
	Reverse	5'-GTCACTGCTGCTGGTGG-3'	
SMα	Forward	5'-GTGAAGAGGAAGACAGCACAGC-3'	384
	Reverse	5'-CTGTCAGCAGTGTCGGATGC-3'	
β-actin	Forward	5'-TCCTGTGGCATCCATGAAAC-3'	314
	Reverse	5'-GAAGCACTTGCGGTGCAC-3'	
